# The use of intracorneal ring segments in keratoconus

**DOI:** 10.1186/s40662-016-0040-z

**Published:** 2016-03-15

**Authors:** Alfredo Vega-Estrada, Jorge L. Alio

**Affiliations:** Keratoconus Unit, Vissum Alicante, Calle Cabañal, 1 Edificio Vissum, 03016 Alicante, Spain; Division of Ophthalmology, Universidad Miguel Hernández, Alicante, Spain

**Keywords:** Keratoconus, Corneal ectasia, Intracorneal rings

## Abstract

Keratoconus is a corneal degeneration that usually appears during puberty and may seriously deteriorate the quality of life of the patients. This corneal disease is today the first indication of corneal transplantation in young patients. Until the last decade of the XX century, keratoplasty procedures were the only alternative to treat this pathological condition. In the beginning of the XXI century, intracorneal ring segments implantation was proposed as a therapeutic choice for treating keratoconus patients. Since then, several published articles have reported the benefits of this surgical procedure in treating this type of corneal ectatic disorder.

The purpose of the present investigative work is to summarize the characteristic of the intracorneal ring segments and also to review the different features published in the literature in relation to this surgical technique for the treatment of keratoconus patients.

## Background

Keratoconus is a progressive corneal ectatic disease characterized by alterations in the morphology of the tissue, which negatively impacts the patient’s visual function and optical quality [1]. Nowadays, there are several therapeutic choices for the management of this condition, such as contact lens wearing, thermokeratoplasty procedures, corneal collagen cross-linking (CXL), intracorneal ring segment (ICRS) implantation, and lamellar and penetrating keratoplasty [2–6].

ICRS are small devices made of synthetic material, which are implanted within the corneal stroma in order to induce a change in the geometry and the refractive power of the tissue. Prof. Joseph Colin proposed the use of such a medical device for the treatment of keratoconus for the first time in the year 2000 [4]. Nevertheless, the idea of implanting a corneal ring to change the refractive power of the cornea was introduced by Blevatskaya in 1966 [7]. The first intracorneal ring design was composed of a 360° ring that led to several complications after the surgery like wound healing-related problems at the incision site; it was the main reason to abandon the 360° ring design and change it for the ring segments that we use today. During the ´80s and in the beginning of the ´90s, the design of ring segments was extensively investigated as an alternative for the correction of refractive errors, specifically myopia. In 1996, Intacs Technology, one of the first companies that designed the ICRS, received the CE certification and later in 1999, the FDA approval for the correction of low to moderate myopia. In spite of the success of ICRS for the correction of such refractive error, this technology was overcome by the good results and popularity of corneal excimer laser procedures.

By this time, Colin and his co-workers observed that ICRS were able to flatten the central cornea and regularize the asymmetry of the tissue, thus leading to a reduction in the keratometric readings and an improvement in the refraction and vision of keratoconus patients. Since then, several authors have reported the benefit of using ICRS in keratoconic eyes with the added value of delaying or avoiding more complex interventions like keratoplasty procedures.

The purpose of the present review is to provide an update of the different features of ICRS implantation as a therapeutic option in the treatment of patients with keratoconus.

## Review

### Types of intracorneal ring segments

**Fig. 1:**
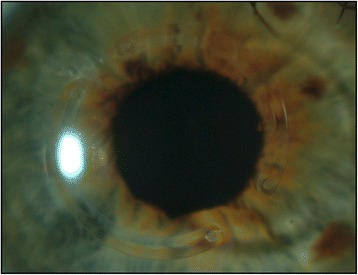
Intracorneal ring segment Keraring (Mediphacos)

**Fig. 2:**
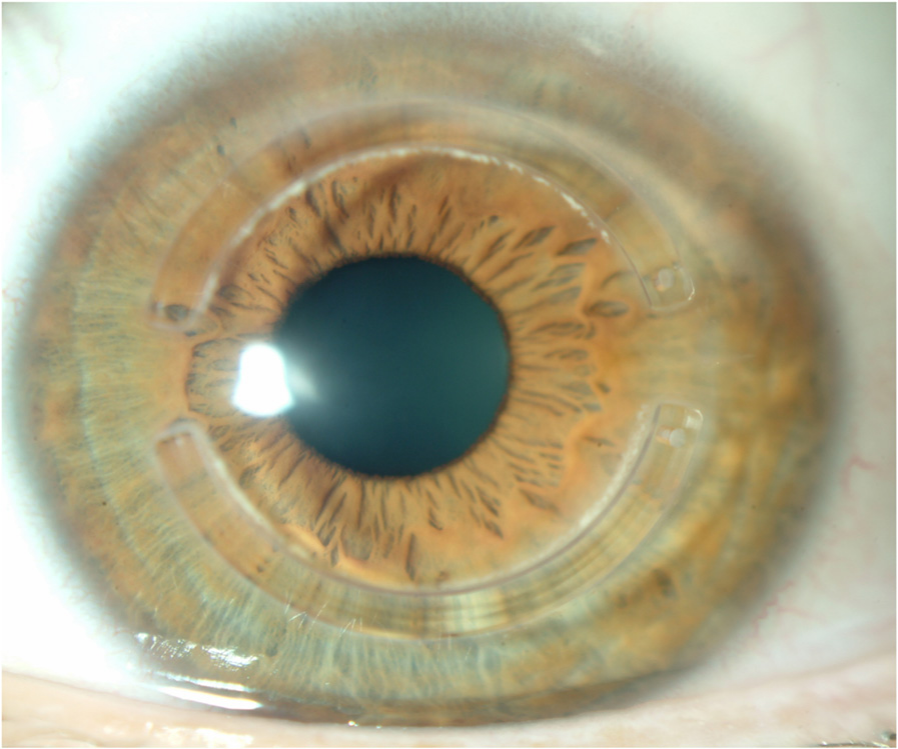
Intracorneal ring segment Intacs (Addition technologies)

**Fig. 3:**
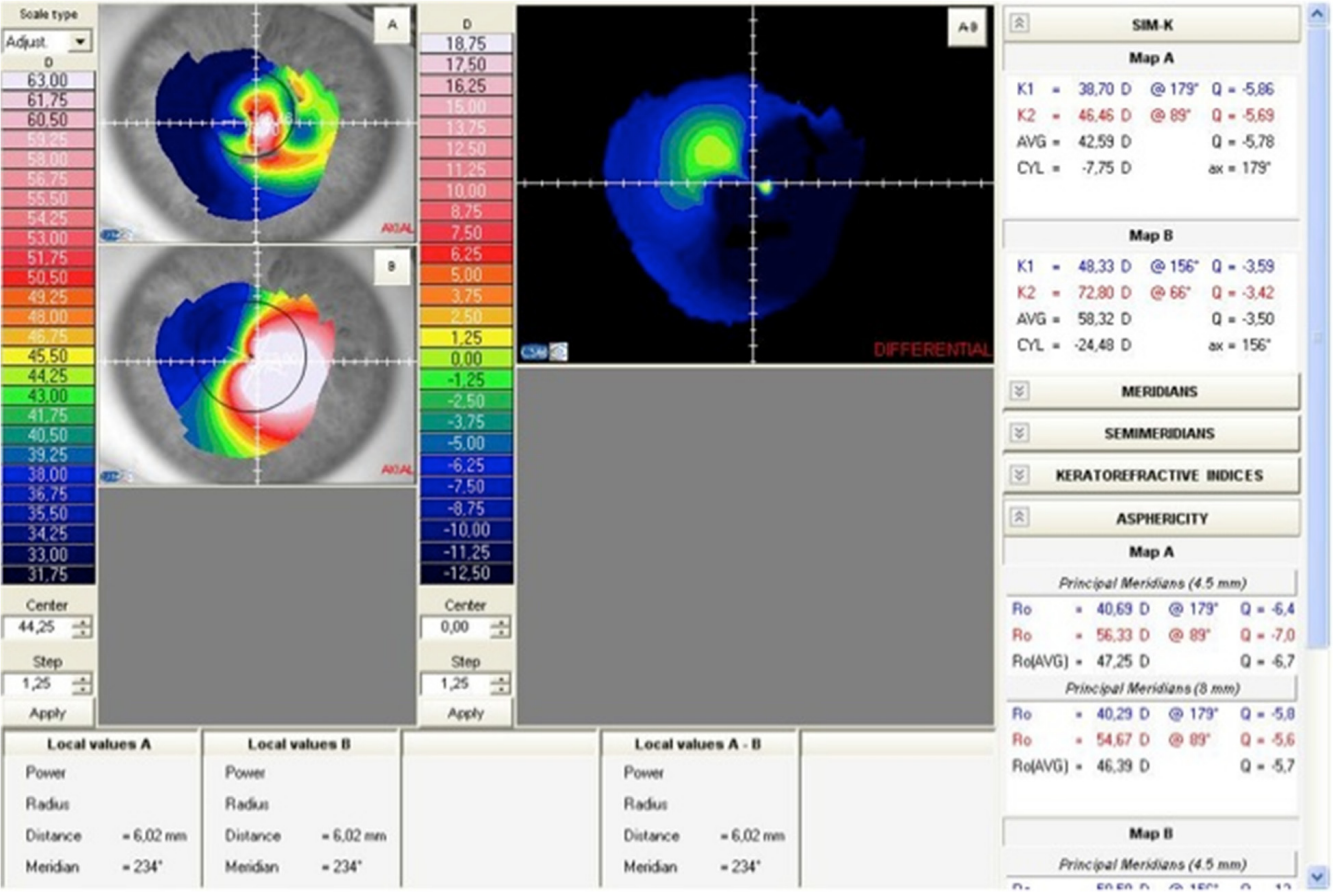
Topography of a patient implanted with a Myoring (Dioptex) showing the significantly flattening that is observed in the postoperative period: Map A: postoperative topography showing an average SimK of 58.32 D, Map B: preoperative topography showing an average SimK of 42.59 D)

Nowadays, there are different types of ICRS commercially available, but the ones that are used more in the clinical practice are Keraring (Mediphacos) (Fig. 1) and Intacs (Addition technologies) (Fig. 2). Table 1 summarizes the main characteristics of these ICRS. In addition, there are two other types of ICRS that because of their smaller diameter and different design, pose more flattening capabilities and are therefore kept for those keratoconic cases that present high myopic refractive errors, the Intacs SK (Addition technologies) and the Myoring (Dioptex) (Fig. 3). The latter is the only device with a 360° ring design with published clinical data. The characteristics of these two types of ICRS are shown in Table 2.

**Table 1:** Intracorneal ring features

Design	Intacs	Kerarings
Arc length (degrees)	150°	90°–210°
Cross section	Hexagonal	Triangular
Thickness (mm)	0.25–0.35	0.15–0.35
Inner diameter (mm)	6.77	5.00
Outer diameter (mm)	8.10	6.00

**Table 2:** Intracorneal ring features

Design	Intacs SK	Myoring
Arc length (degrees)	150°	360°
Cross section	Oval	Triangular
Thickness (mm)	0.40–0.45	0.15–0.35
Inner diameter (mm)	6.00	5.00–8.00
Outer diameter (mm)	7.00	5.00–8.00

In recent years, Mediphacos developed an interrupted ring of 355°, which is available in a diameter of 5.7 mm and a thickness ranging from 200 to 300 μm. Although there are no follow up longitudinal studies reporting results with this type of ring, the few investigations published in the literature show an improvement in the visual and refractive status of patients with central keratoconus [8]. Nevertheless, this type of ring design reported complications, such as corneal melting, extrusion of the segment, and alterations of the tissue at the incision site [9]. Therefore, a further evaluation with a longer period of follow up should be conducted in order to assess the potential complications that may be found with this type of ring design.

### Mechanism of action of the ICRS

ICRS act as spacer elements between the collagen fibers of the corneal tissue [10]. This way, ICRS will induce an arc-shortening effect thus flattening the central area of the cornea. Some theoretical models based on finite element analysis have shown that the flattening effect observed after ICRS implantation is directly proportional to the thickness of the segment and inversely proportional to the corneal diameter where it is implanted. This means that the thicker the segment and the smaller the diameter in the cornea where the device is implanted, the higher the flattening effect will be achieved. [11]. Nevertheless, these theoretical analyses apply only to normal corneas where there is an orthogonal arrangement of the collagen fibers. In patients with keratoconus, this special disposition of the fibers is lost, which leads to a more unpredictable response of the segments in these types of corneas [12]. Another theory that may explain the mechanism of action of the ICRS is the “Thickness law” proposed by Barraquer that states that when tissue is added to the periphery of the cornea or tissue is removed from the center, a flattening of the cornea will be achieved and vice versa [13]. However, there is not enough scientific data published in the literature to support this theory.

### Surgical procedures

With the purpose of implanting the ICRS deep into the cornea, we need to perform channels in the stroma where the segments will be inserted. For this purpose, there are two different surgical interventions: mechanical and femtosecond laser assisted technique.

In the mechanical or manual technique, the surgeon must mark the center of the pupil in order to use it as a reference point during the procedure. Then, a calibrated diamond knife is used to create an incision at a depth of 70 % of the corneal pachymetry. A suction ring is placed around the corneal limbus in order to fixate the eye during the dissection of the corneal stroma. Two semicircular dissectors are then placed through the incision and advanced in the deep stroma in a clockwise and counter-clockwise movement aiming to perform a tunnel within the corneal lamellas.

The other technique used to create the tunnels is with the femtosecond laser. In this case, a coupling interface is place over the cornea with a disposable device, which allows a precise focus of the laser beam, thus creating a dissection at the desired depth. The tunnel is then created at approximately 70 or 80 % of the corneal pachymetry without directly manipulating the eye. Finally, the ICRS are inserted in the created tunnels.

### Implantation nomograms

In order to decide the number, arc length, thickness, and position of the segments in the cornea, we use a clinical guideline that is known as implantation nomogram. Even though several authors have reported good results when implanting ICRS in keratoconic eyes, the main limitations that nomograms have are that most of them are based on anecdotic clinical data or variables that are very subjective in patients with keratoconus, such as spherocilyndrical refraction and topographic pattern of the cone. In relation to the number or segments to be implanted, some authors found that implanting a single ring segment will provide better results when comparing the outcomes of those cases where two segments were implanted [14]. On the contrary, in an investigation conducted by our research group it was found that based on the topographic pattern of the keratoconus, the best choice was to implant one segment in those cases of inferior steepening and two segments in central cones [15]. In addition, other investigations proposed using 2 segments of 160° arc length depending on the spherical equivalent to be corrected in keratoconic patients [16]. Authors of this work specifically used a different thickness on the segment depending on the severity of the disease. They observed that the best results were obtained when implanting 2 segments of 200 μm thickness for the correction – 2 diopters (D), 250 μm to correct −4 D, 300 μm to correct −6 D, and 350 μm to correct −8 D. Other authors have obtained good results using the same nomogram with the implantation of a ring segment of 120° of arc length [17]. Siganos and co-workers also proposed a similar approach [18] – this group obtained good results when implanting 2 segments of 160° arc length and changing the segments thickness in the following manner: 150 μm for those keratoconic cases of less than 4 D of myopia; 200 μm to correct between −4.25 and −6 D; 250 μm to correct −6.25 and −8 D; 300 μm for −8.25 to −10 D, and 350 μm for those cases with more than 10 D of myopia.

Regarding the location of the segments, there are some authors who claim that the best location to implant the segments is by placing the corneal incision in the temporal site of the cornea [17, 19–21] or in the steepest meridian of the cornea [22, 23]. There are other works that have reported good results when implanting the ICRS guided by the comatic axis [24]. Recently, our research team published work in which we concluded that the best outcomes for implanting ICRS were observed in those cases where the refractive and topographic cylinder did not differ in more than 15° of separation [25].

There are different approaches regarding the guidelines to be used when implanting ICRS. Nevertheless, today the most widespread nomograms used in the clinical practice are those developed by the main manufacturers of ICRS.

### Results of ICRS implantation

Since Colin reported for the first time the results of ICRS implantation for the treatment of keratoconus in the year 2000 [4], several authors have demonstrated the efficacy of this surgical technique in reducing the spherical equivalent and keratometric readings in patients with keratoconus [21–25]. Most of these studies report an improvement in the uncorrected and corrected visual acuity as well as in the spherical equivalent and in the cylinder. The majority of the authors observed a central flattening of the cornea that was consistent with a mean reduction of the keratometric readings between 3 and 5 D [18, 26–29]. Additionally, studies that have assessed the optical quality by analyzing the changes in anterior corneal higher order aberrations, have found a reduction in these parameters after ICRS implantation, specifically in the asymmetric aberrations (coma and coma-like). These changes observed in the aberrometric coefficient are expected to occur due to the capability of the implants in regularizing the geometry of the corneal tissue [29–31].

Even when most authors have reported good results in terms of improvement in visual acuity, a recent multicentric study performed by our research team found that the efficacy of ICRS implantation was related to the visual limitation of the patients at the time of surgery [29]. In the aforementioned investigation, the outcomes of the surgical procedure were analyzed based on a grading system that takes into account the visual acuity of the patients diagnosed with keratoconus [32]. We observed that those patients with good visual function at the time of surgery were more prone to lose lines of vision after the procedure. On the other hand, those cases with severe visual impairment before the procedure were the ones that benefited the most from ICRS implantation [29] (Table 3). These findings led us to consider that ICRS implantation in cases with keratoconus and good vision should be undertaken with extreme caution because of the risk of losing vision in this group of patients.

**Table 3:** Percentage of patients that gain or lose corrected vision after ICRS implantation

Visual Acuity	Gain ≥ 1 line CDVA	Lost ≥ 1 line CDVA	Lost ≥ 2 lines CDVA
CDVA ≥ 0.6 GRADE I + II	37.90 %	36.29 %	25.80 %
CDVA ≤ 0.4 GRADE IV + PLUS	82.85 %	10.00 %	4.28 %

*CDVA=* corrected distance visual acuity, *ICRS=* intracorneal ring segment

Long-term outcomes of ICRS implantation for the treatment of keratoconus have always been a topic of debate. There are some studies published in the literature that hypothesized that the distribution of the forces along the stroma that is observed after the implant may help in reducing the stress on a specific point of the tissue, thus leading to a more biomechanically stable cornea [33]. Nevertheless, these observations have not been completely proven in the clinic. Even though there are some long term studies that have reported the stability of the surgical procedure [22, 31, 34], there is a clear limitation in most of these reports as they do not specify if the type of patients that they were evaluating within their cohort belonged to cases with the progressive or stable form of the disease, or if they just analyzed patients with stable keratoconus. In a recent study carried out by our research group, it was observed that long-term stability of ICRS implantation depended on the progression pattern of keratoconus at the time of surgery. Thus, in those cases with the stable form of the disease, ICRS implantation does not cause significant changes after a long period of follow up [31]. Nevertheless, in those cases that show clinical signs of progression, the benefit achieved immediately after the procedure is expected to be lost after a long period of time. From that study, we concluded that the stability of the disease should be confirmed before suggesting ICRS implantation in keratoconic patients [35].

### Combined procedures

Keratoconus is an ectatic corneal disorder characterized by progressive corneal thinning and morphological alterations of the tissue often accompanied by refractive errors. ICRS implantation is a surgical technique that has demonstrated improvements in the morphological alterations of the cornea. Nevertheless, it shows lack of accuracy in the refractive predictability and its capability in halting the progression of the disease is often controversial. For this reason, it seems logical to think that combining therapeutic approaches will improve the different aspects of the disease in keratoconic patients.

In order to stop the progression that is observed in patients with corneal ectatic disorder, several authors have demonstrated that the best option is to perform CXL [36–38]. In addition, there are some reports published in the scientific literature that have shown that a combination of ICRS and CXL improves the vision and the refraction of patients with keratoconus [39, 40]. Moreover, there are some investigations reporting that a combination of ICRS together with CXL leads to more flattening of the cornea and reduction of the corneal cylinder than those cases treated with ICRS alone [41]. Additionally, in one of the few prospective, randomized investigations published in the literature, Coskunseven et al. demonstrated that the sequence ICRS followed by CXL lead to better visual, refractive and topographic outcomes when compared with those patients operated with CXL and then with ICRS [42]. However, in a recent study comparing ICRS alone and combined with CXL, the authors concluded that there were no significant differences between the two approaches [43].

In order to reduce the refractive error that is often present in patients with keratoconus, some authors have proposed the use of ICRS and CXL together with photorefractive keratectomy (PRK). Although there are just a small number of studies in the scientific literature analyzing this therapy, Lovieno and co-workers reported good visual, refractive and aberrometric outcomes when treating keratoconic eyes with ICRS, CXL, and PRK on the same day [44]. In a similar fashion, Kremer et al. observed good results when performing CXL and PRK in patients with keratoconus who were previously treated with ICRS [45].

As shown in the different investigations mentioned above, ICRS may be successfully combined with other therapeutic approaches, such as CXL or PRK, in order to improve the visual, refractive and keratometric parameters in patients with keratoconus. Nevertheless, the sequence of the treatments or whether to perform them together in the same surgical session is still a topic of controversy.

### Indications

Selecting the adequate patient for ICRS represents an important challenge for the clinician when facing a therapeutic approach in a keratoconic patient. A full ophthalmic examination should be performed including the following: 1) corrected and uncorrected visual acuity; 2) corneal topography including corneal aberrometry; as most patients with keratoconus wear contact lenses, discontinuation must be advised at least 2 weeks prior to the examination in those cases where soft contact lenses are used and 1 month in those cases wearing rigid contact lenses, in order to increase the reliability of the examination; 3) corneal pachymetry, preferably a corneal pachymetric map aiming to assess the appropriate thickness of the site of ICRS implantation 4) corneal biomechanics, either ocular response analyzer or Corvis ST.

Most authors in the scientific literature agree with the following indications:

- Corrected distance visual acuity < 0.9 in the decimal scale.
- Intolerance to contact lens use
- Absence of central leucoma

### Complications

**Fig. 4:**
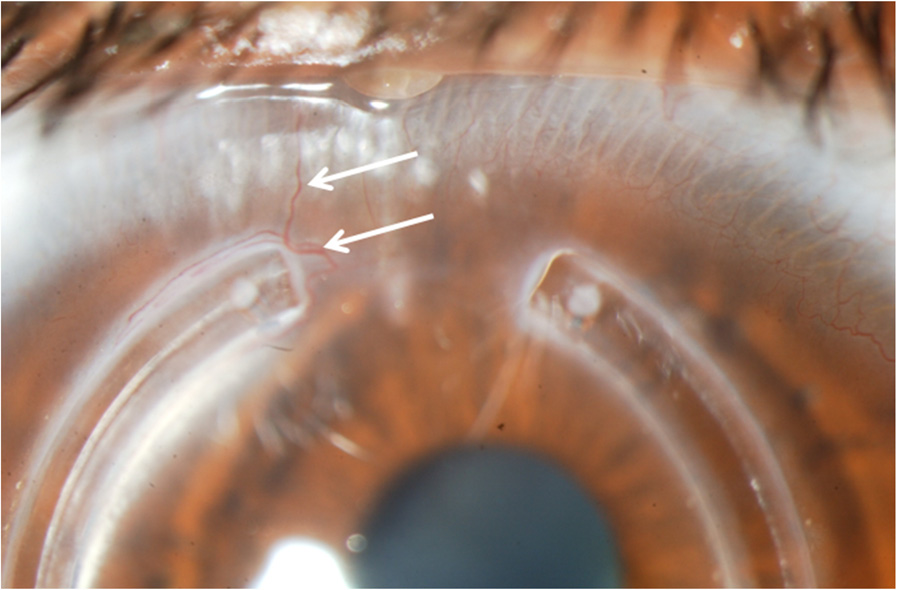
Neovascularization of an ICRS

Implanting ICRS in keratoconic patients is considered to be a safe surgical procedure mainly due to the advent of femtosecond technology that provides more precise and predictable size and depth of the stromal tunnels. Although rare, intraoperative complications have been described when performing the channels with the manual technique. The intraoperative complications that are more often observed are segment decentration, inadequate depth of the tunnels, and asymmetry of the segments [16]. In relation to the postoperative complications, we can observe: ring segment extrusion, corneal neovascularization (Fig. 4), corneal haze, segment migration, corneal melting, and infectious keratitis, among others. One of the most frequently observed findings is white deposit around the segment inside the tunnels. Histopathological analyses have demonstrated that the deposit corresponds to fatty acids and do not interfere with the visual function of the patient or morphology of the corneal tissue [46].

One of the main advantages that ICRS implantation has is its reversibility. Even when some of the above-mentioned complications might appear, some studies have shown that segment explantation can be safely performed with visual, refractive and topographic variables coming to preoperative levels [47].

## Conclusions

To conclude, we consider ICRS being one of the most effective treatment alternatives in the management of keratoconus patients. It is a safe and reversible technique, which regularizes the morphological alterations present in the cornea, thus improving the visual function and the quality of life of patients with keratoconus. The stability of the results will depend on the progressive nature of the disease at the moment of the surgery; this way, ICRS provides long-term stability of the outcomes in those patients with no clinical signs of progression. There are still some studies that should be performed in order to analyze the outcomes of this surgical technique depending on the severity of the disease. In addition, the biomechanical behavior of ICRS implantation and the potential effect on the tissue is not completely understood and further investigations assessing this topic are needed. Finally, the nomograms of implantation are currently based on empirical data and the subjective analysis of the clinician; thus we need new mathematical and scientific based models that provide a more objective guideline for ICRS implantation in patients with keratoconus.
